# Fertility care amidst the COVID19 pandemic: the American experience

**DOI:** 10.1186/s13048-021-00782-4

**Published:** 2021-02-18

**Authors:** Austin D. Schirmer, Jennifer F. Kawwass, Eli Y. Adashi

**Affiliations:** 1 Emory Reproductive Center, Department of Gynecology and Obstetrics, Emory University School of Medicine, Atlanta, USA; 2 Department of Medical Science, the Warren Alpert Medical School of Brown University, Providence, USA

## Abstract

The COVID-19 pandemic has claimed the lives of over one million people worldwide, and has affected all aspects of healthcare worldwide, including the delivery of care to patients with fertility-related diagnoses. In the United States, the response of US fertility clinics to the COVID-19 pandemic was coordinated by the *American Society for Reproductive Medicine* (ASRM). ASRM acted quickly to develop guidelines for limiting fertility treatment and clinic consultations during the early days of the pandemic, and then safely restarting fertility treatment. A survey of patients with fertility-related diagnoses who presented for care during the first months of the pandemic revealed that a majority of patients who presented for care during the early months of the pandemic experienced delayed or cancelled treatment cycles. Patients with infertility subsequently reported a desire to resume fertility care, but emphasized the importance of their clinic having policies and procedures in place to limit the risk of infection.

## Commentary

In early December 2019, the first severe cases of pneumonia caused by the novel coronavirus COVID-19 were beginning to emerge in Wuhan, China, thereby marking the beginning of a global pandemic that to date has claimed the lives of over two hundred thousand people in the US and over one million people worldwide [1, 2]. The COVID-19 pandemic has also had unprecedented effects on the delivery of healthcare and on the ability to treat a broad range of acute and chronic medical conditions, including the treatment of patients with fertility-related diagnoses. Treatment of infertility and other fertility-related conditions is time-sensitive, yet rarely considered urgent, with the exception of patients wishing to cryopreserve gametes prior to gonadotoxic therapy. Once the severity of the COVID-19 pandemic was realized, reproductive endocrinologists and their professional societies throughout the world were forced to rapidly make the unprecedented decision to delay or severely limit treatment with an eye toward protecting patients and staff and avoiding further burdening of a stressed healthcare system with non-urgent medical treatment. In the US, this response was coordinated by the *American Society for Reproductive Medicine* (ASRM).

The ASRM acted quickly in response to the developing global pandemic by releasing its initial recommendations before the most severe effects of COVID-19 were felt in the US. To this end, the ASRM assembled a COVID-19 task force tasked with developing official guidance for US fertility clinics. It was on March 17th that the ASRM issued guidelines intended to prevent healthcare-acquired infections and to limit the use of healthcare resources on non-urgent treatment. These guidelines recommended suspending the initiation of new fertility treatment cycles, including intrauterine insemination (IUI), *in vitro* fertilization (IVF*)*, and non-clinically urgent gamete cryopreservation. Given the paucity of peer-reviewed data regarding COVID-19 and pregnancy outcomes, the ASRM further recommended the cancellation of all planned embryo transfers, whether fresh or frozen. The guidelines did not call for the cancellation of IVF cycles that had already been started. Clinically-urgent gamete cryopreservation, such as in patients with cancer or other conditions requiring gonadotoxic therapy, remained permissible when safe and feasible. The guidelines further called for the suspension of elective surgeries and of non-urgent diagnostic procedures, which mirrored guidance provided by the US Surgeon General to US hospitals and surgery centers. Finally, the initial guidance of the ASRM called for in-person consultations and interactions to be minimized while emphasizing that telemedicine should be used as a substitute whenever possible [3].

The ASRM COVID-19 task force reconsidered its guidance regarding fertility treatment during the pandemic on March 30th, 2020. However, no new guidelines were issued until the end of April 2020. On April 13th, the ASRM, while maintaining its position regarding the suspension of most fertility treatment, announced the formation of a diverse subgroup, comprising academic and private practice physicians, advisors, and external experts to draft guidelines for resuming fertility care. This task force was charged with prioritizing the health and safety of patients, physicians, and clinic staff, while also considering the time-sensitive nature of fertility treatment and the impact of delayed treatment on the prognosis of patients with fertility-related conditions. While developing guidelines for safely resuming fertility care, this task force also considered the variable impact of the COVID-19 pandemic on different geographical locations throughout the US, the varying availability of COVID-19 viral antigen and antibody testing, the availability and utilization of healthcare supplies and resources critical to hospitals and other healthcare facilities, and federal, state, and local government regulations that might impact the ability of fertility clinics to render safe and effective treatment [4].

Based on input from the subgroup formed in mid-April, the ASRM published guidelines for when to resume fertility care, how to assess the risk of resuming care, and how to mitigate these risks. Emphasizing the need for healthcare providers to assess local conditions affecting the safety and impact of resuming care on their local community, the ASRM recommended that, in general, several milestones should be considered when resuming care. These milestones included a sustained regional reduction in COVID-19 cases in the vicinity of the clinic, and the ability of local hospitals to safely treat all patients without resorting to crisis-standards of care [5]. Fertility practices were also advised to perform a formal, documented assessment of risk, in which considerations such as the ability of the practice to mitigate the risk of COVID-19 infection, and the possibility of causing permanent, negative consequences for their patients by delaying fertility care should be included.

 To mitigate the risks associated with resuming fertility care, the ASRM recommended that clinics develop and implement written, comprehensive policies addressing staff and patient care. According to this guidance, which included information published by the *Occupational Safety and Health Administration* (OSHA) and the *Centers for Disease Control and Prevention* (CDC) for mitigating risk in healthcare and workplace settings, clinics were advised to craft policies to limit risk to staff members who might be particularly susceptible to developing severe illness from a COVID-19 infection. Clinics were also advised to develop written sick leave policies for staff, and to consider implementing symptom/temperature screening, handwashing, and facemask wearing policies. Frequent handwashing and surface decontamination was emphasized. Importantly, this guidance also specified that symptom and temperature screening as well as mask wearing policies should apply to patients as well as to clinic staff. The guidelines further emphasized that personal protective equipment (PPE) should be kept in sufficient supply and that clinic staff should be trained regarding appropriate PPE use for a given clinic setting.

The ASRM guidelines also acknowledged the adverse impact that delaying patient care could have on specific patient populations and recommended considering medical, psychological, and emotional factors in prioritizing which patients to offer care to first when resuming care. These considerations included advanced patient age, diminished ovarian reserve, the presence of known or suspected endometriosis, the mental health and emotional wellbeing of patients, and the impact of delayed care on a patient’s ability to access treatment due to insurance coverage or employment status. Other considerations, such as the number of clinic visits required for specific treatment plans, were incorporated as well in that treatments that required few clinic visits to complete were considered safer.

The ASRM further emphasized the need to limit the number of patients coming into to clinic to the extent possible when resuming care. Recommendations addressing this facet of care focused on implementing and using telemedicine whenever possible for consultations, allowing clinic staff to work from home to the extent possible, limiting monitoring visits to the minimum necessary to safely complete treatment, and, importantly, limiting the number of visits for which a patient’s partner would be allowed to accompany the patient [6].

Although the ASRM, as an organization, has broad influence on the practice of reproductive endocrinology in the US, compliance with the ASRM guidelines by fertility clinics is not compulsory. It is indeed difficult to know what proportion of US fertility clinics followed the ASRM guidelines regarding fertility treatment during the early phases of the COVID-19 pandemic. Preliminary prospective reporting data from the SART-CORS dataset suggests that over the course of 2020, the total number of IVF cycles approximated those in 2019, suggesting that while the pandemic paused treatment for a period of time, the cycles were most likely delayed, not cancelled [7]. A survey performed by the company FertilityIQ may provide some early insight. In their survey of 1,808 women in the US, the majority of whom self-identified as having infertility, 63.0 % of respondents indicated that their clinic had delayed or cancelled their treatment cycle, 23.3 % indicated that they had decided to delay or cancel their own treatment cycle, and 13.7 % indicated that their treatment had continued as planned during the early months of the pandemic. The respondents also indicated in the survey, which was performed after the ASRM guidance for safely resuming treatment was released, that 44.5 % had resumed treatment. Of those who had not resumed treatment, 36.7 % indicated that they had chosen to further delay their treatment, while the rest cited either continued clinic closure, or a clinic-imposed delay.

According to the FertilityIQ survey data, in response to the question “how do you feel about undergoing treatment at this time in light of the COVID-19 oubtreak,” 45.6 % of patients answered that they felt “comfortable” undergoing treatment. However, the respondents had strong feelings regarding the infection control policies of their clinics. An overwhelming majority of patients indicated that they considered compliance, by their clinic, with the ASRM guidelines to be either highly (65.4 % of respondents) or somewhat (29.4 % of respondents) important to them. The majority (51.7 %) of respondents indicated that it is very important that their clinic offer telehealth visits, and most respondents (86.2 %) indicated that they considered it highly important that the clinic staff wear masks and gloves when rendering care. Similarly, a large proportion (76.2 %) of respondents felt that clinic policies requiring patients to wear a cloth mask were highly important. While compliance with the ASRM recommendations and PPE use proved of import to patients, many patients found clinic policies that prevented their partner from attending certain procedures to be either highly concerning (45.9 %) or somewhat concerning (21.5 %), suggesting that this policy, in particular, was a source of stress for patients. Although many patients have resumed fertility treatment, many expressed concern regarding the safety of becoming pregnant during the pandemic, with 25.8 % indicating that they found the risks of being pregnant during the COVID-19 pandemic to be very concerning, and 52.9 % indicating that they found these risks to be somewhat concerning [8].

## Conclusion

The response of ASRM to the COVID-19 pandemic was swift and decisive, and likely prevented healthcare-acquired infections in patients, physicians, and clinic staff while preserving precious healthcare resources for use in managing the initial response to COVID-19 in the US. These actions, though necessary, will undoubtedly have long-term medical, psychological, and financial effects on patients with infertility. Data from the *Society for Assisted Reproductive Technology Clinical Outcome Reporting System* (SART CORS) and the *National ART Surveillance System* (NASS) maintained by the CDC will, in time, elucidate the extent to which fertility clinics limited or stopped treatment during the pandemic, as well as the rate at which couples with infertility in the US resumed treatment during and after the pandemic. The effects of the pandemic on the long-term outcomes and mental health of couples with infertility who had planned to start treatment when COVID-19 emerged, however, will be harder to assess, and are an important subject for research in the coming years [4]. The recent experiences of fertility clinics in the US and worldwide has cast in sharp relief the potential for a global pandemic to disrupt normal operations and adversely impact the delivery of safe, effective care. While recent efforts by the American Society for Reproductive Medicine and fertility clinics in the US have, of necessity, been focused on responding to the current pandemic, there is a clear need for national guidelines establishing measures that clinics should take to prepare for future pandemics. Ongoing and future research on the impact of the COVID-19 pandemic is critical to this process, as the lessons learned during COVID-19 will serve as an important foundation for developing measures to limit the adverse impact of similar events in the future.

## Data Availability

The data from the survey that was conducted by FertilityIQ was provided by FertilityIQ. It is available upon reasonable request.

## Abbreviations

ASRM: American Society for Reproductive Medicine
IVF: *In vitro fertilization*
OSHA: Occupational Health and Safety Administration
CDC: Centers for Disease Control and Prevention
PPE: Personal protective equipment
SART CORS: Society for Assisted Reproductive Technology Clinical Outcome Reporting System
NASS: National ART Surveillance System

## References

[1] Guan W-j, Ni Z-y, Hu Y, Liang W-h, Ou C-q, He J-x (2020). Clinical Characteristics of Coronavirus Disease 2019 in China. N Engl J Med.

[2] WHO Coronavirus. Disease (COVID-19) dashboard. [cited 2020 September 27th, 2020]; Available from: https://covid19.who.int/.

[3] American Society for Reproductive Medicine (ASRM) Patient Management and Clinical Recommendations During The Coronavirus (COVID-19) Pandemic. 2020 March 17th, 2020 [cited; Available from: https://www.asrm.org/globalassets/asrm/asrm-content/news-and-publications/covid-19/covidtaskforce.pdf.

[4] American Society for Reproductive Medicine (ASRM). Patient Management and Clinical Recommendations During The Coronavirus (COVID-19) Pandemic: Update #2. 2020 April 13th, 2020 [cited; Available from: https://www.asrm.org/globalassets/asrm/asrm-content/news-and-publications/covid-19/covidtaskforceupdate2.pdf.

[5] American Society for Reproductive Medicine (ASRM) Patient Management and Clinical Recommendations During The Coronavirus (COVID-19) Pandemic. Update #3. 2020 April 24th, 2020 [cited; Available from: https://www.asrm.org/globalassets/asrm/asrm-content/news-and-publications/covid-19/covidtaskforceupdate3.pdf.

[6] American Society for Reproductive Medicine (ASRM). Patient Management and Clinical Recommendations During The Coronavirus (COVID-19) Pandemic: Update #4. 2020 May 11th, 2020 [cited; Available from: https://www.asrm.org/globalassets/asrm/asrm-content/news-and-publications/covid-19/covidtaskforceupdate4.pdf.

[7] Wantman E. (personal communication, September 30th, 2020).

[8] Survey of Patients. with Infertility about COVID-19. FertilityIQ - unpublished data; 2020.

